# Maternal Anxiety, Infant Stress, and the Role of Live-Performed Music Therapy during NICU Stay in The Netherlands

**DOI:** 10.3390/ijerph18137077

**Published:** 2021-07-02

**Authors:** Karianne E. Kraft, Artur C. Jaschke, Anne-Greet Ravensbergen, Annet Feenstra-Weelink, Maud E. L. van Goor, Marlou L. A. de Kroon, Sijmen A. Reijneveld, Arend F. Bos, Nienke H. van Dokkum

**Affiliations:** 1 Division of Neonatology, Department of Pediatrics, Beatrix Children’s Hospital, University Medical Center Groningen, University of Groningen, Hanzeplein 1, 9713GZ Groningen, The Netherlands; a.c.jaschke@umcg.nl (A.C.J.); a.g.ravensbergen@umcg.nl (A.-G.R.); j.feenstra@umcg.nl (A.F.-W.); m.e.l.van.goor@gmail.com (M.E.L.v.G.); a.f.bos@umcg.nl (A.F.B.); n.h.van.dokkum@umcg.nl (N.H.v.D.); 2 Department of Music Therapy, ArtEZ University of the Arts, Hulsmaatstraat 35, 7523WB Enschede, The Netherlands; 3 Department of Health Sciences, University Medical Center Groningen, University of Groningen, Hanzeplein 1, 9713GZ Groningen, The Netherlands; m.l.a.de.kroon@umcg.nl (M.L.A.d.K.); s.a.reijneveld@umcg.nl (S.A.R.)

**Keywords:** music therapy, neonatal intensive care unit (NICU), maternal anxiety, preterm infants, infant stress

## Abstract

Having an infant in the neonatal intensive care unit (NICU) elicits maternal anxiety, which may hamper parent−child bonding. We performed a prospective cohort study to describe anxiety in mothers of infants born before 30 weeks of gestation during NICU stay in The Netherlands, and investigated the influence of infant stress and gestational age. Second, we performed a randomized-controlled live-performed music therapy trial (LPMT trial) to investigate whether music therapy applied to the infant alleviated maternal anxiety. The relation between infant stress, gestational age, and maternal anxiety was measured in 45 mother−infant dyads, using the Neonatal Infant Stressor Scale and the State-Trait Anxiety Inventory (STAI). The effect of LPMT on anxiety was assessed in 21 mothers whose infants were assigned to either LPMT (*n* = 12) or waitlist (*n* = 9). Mothers completed the STAI before and after this period. Maternal anxiety decreased over time in all mothers, and was strongly related with infant stress (r = 0.706, *p* < 0.001), but not with gestational age. Anxiety scores decreased by 12% after LMPT, and increased by 1% after a waitlist period (*p* = 0.30). Our results indicate that LPMT in the weeks after birth may accelerate the reduction of maternal anxiety. Further research should focus on the effects on mother−child bonding.

## 1. Introduction

Both parents and their sick or preterm infants frequently experience a stay in the neonatal intensive care unit (NICU) as a stressful period [1,2]. For infants, stress can be evoked by the NICU environment, including noise, light, and separation from parents [2], and by exposure to life-saving, but painful or stressful, interventions, such as heel lancing for blood tests, nasal or oral tube insertion, long-term ventilation, and major surgery [3]. There is increasing evidence that early neonatal stress exposures also can affect long-term neurodevelopmental outcome in preterm-born children [2,3,4].

For parents of very preterm infants, the alteration in their parental role is recognized as the most important stressor [5,6]. This altered role may contribute to an increased risk of feeling depressed or anxious, compared with parents of healthy full-term infants [7]. Parents may feel disrupted in their ability to care for the infant, because of illness severity, medical procedures they cannot perform, and limited access [5,6]. Whether a relationship exists between the stress experienced by an infant and the anxiety subsequently experienced by its parents has not been demonstrated before, even though this seems obvious. A recent meta-analysis on parental stress in the NICU concluded that mothers reported higher stress levels than fathers [5]. Postpartum maternal stress experienced during the NICU stay is negatively associated with infant growth, (neuro)development, nutrition, and bonding [8]. Moreover, approximately 20% of parents still experience signs of stress one year after a NICU admission of their infant [9], which may further hamper child (neuro)development [8,10,11].

Music therapy is a recent and promising innovation in neonatal care, where efforts are constantly being made to reduce both infant stress and parental anxiety levels. Besides well established early interventions, such as kangaroo care [12], family centered care [13], cognitive behavioral therapy [14], and peer-support programs [15], music therapy is increasingly being implemented. Music therapy can be defined as “the clinical and evidence-based use of music interventions to accomplish individual goals within a therapeutic relationship by a credentialed professional who has completed an approved music therapy program” [16]. Music interventions have been shown to have beneficial effects on infants’ vital signs [17,18,19,20], as well as on preterm movement patterns, which may implicate a positive effect on infant neurodevelopment [21]. More recently, there is growing interest in the potential stress-reducing effect of music-based interventions on parents in the NICU, and its possible value in enhancing the developing relationship between parents and their infants [22]. The limited evidence indicates that music interventions decrease parental stress perception and reduce maternal anxiety symptoms in parents of preterm infants [17,23].

With a better understanding of maternal anxiety during NICU stay and the factors influencing this anxiety, efforts can be made to reduce maternal anxiety as much as possible, which may in turn have major benefits for infant development. Therefore, our first aim was to describe maternal anxiety early during NICU stay and at discharge from the NICU, as well as its relation with infant stress and gestational age. Secondly, we aimed to determine the effects of our music therapy program on maternal anxiety, incorporating infant stress as an additional factor. We hypothesize that mothers experience high levels of anxiety, which decrease during NICU stay, and which is influenced by both gestational age and the infant stress. Moreover, we hypothesize that music therapy reduces maternal anxiety.

## 2. Materials and Methods

### 2.1. Setting and Population

Parents of infants born with a gestational age of less than 30 weeks, who were admitted to the NICU of the University Medical Center Groningen, The Netherlands, were approached to participate in two ongoing prospective studies. They could choose to participate in either one or both of the studies.

The first study regarded a prospective cohort study called “Stress and Outcomes in NICU Graduates (STRONG)”, with the inclusion of infants born between September 2019 and January 2020. This study aimed to investigate infant stress and maternal anxiety and the effects on the epigenetic profiles and neurodevelopment. We used data collected as part of this study on infant stress and maternal anxiety in order to achieve our first aim.

The second study was used to achieve our second aim and regarded an ongoing Live-Performed Music Therapy trial (LPMT trial). We included infants born between August 2019 and March 2021. Infants were randomly assigned to either the intervention of LPMT, or a waitlist for a period of two weeks. Because this intervention could not be provided in a blinded manner, we chose a cross-over design, so that all parent−infant dyads would have the opportunity to receive LPMT during a four-week period. The mother−infant dyad was randomized to start either the intervention or the waitlist period from days 7 to 14 after birth onwards. After randomization, infants in the intervention group received LPMT for a two-week period. Infants in the waitlist group served as the control group. The effects of our LPMT trial were evaluated after this two-week period. For ethical reasons, after this two-week period, infants from the waitlist group also received LPMT for another two-week period. Because of the limited availability of our music therapist, we did not provide LPMT during the second two-week period to the infants that had received LPMT during the first two-week period. Within the second two-week period, infants were mostly discharged to high-care wards in regional hospitals. Both parents were approached in the first week after birth to participate in the LPMT trial. For the current study, we included the questionnaires completed by the mothers.

**Figure 1:**
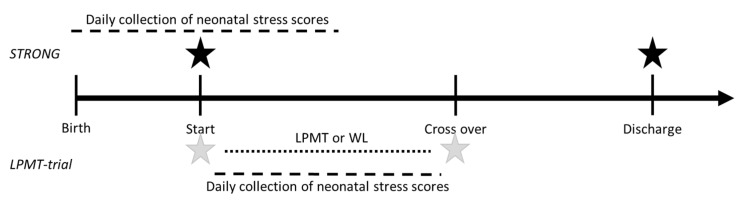
Study procedures of the “Stress and Outcomes in NICU Graduates (STRONG)” and Live-Performed Music Therapy Trial (LPMT trial). Black stars represent the timing of the State and Trait Anxiety Inventory in the STRONG study. Grey stars represent the timing of the State and Trait Anxiety Inventory in the LPMT trial.

For both studies, infants whose parents did not speak or understand Dutch were not eligible for inclusion. Parents were asked to provide written informed consent. Both studies were approved by the Medical Ethical Committee of the University Medical Center Groningen (METc 2019/128 and METc 2019/093) and were registered online (ISRCTN62164166 and ISRCTN94562698). The study procedures are summarized in Figure 1.

### 2.2. Developmental Care Practices at Our NICU

At our NICU, parental presence is highly encouraged, and both mothers and fathers are always welcome to talk to or touch their infant. In close cooperation with the nursing staff, parents are also involved in care as much as possible. Kangaroo care is implemented shortly after birth for at least 60 min daily, even when infants are on mechanical ventilation. We stimulate mothers to breastfeed their preterm infants and offer lactation coaching to support them. LPMT has recently been added to these practices in the research setting of our LPMT trial. During LPMT, parents are allowed to use touch and voice, but simultaneous kangaroo care is not allowed because of possible overstimulation in infants born <30 weeks’ gestation.

### 2.3. Measures and Procedure

#### 2.3.1. Outcome Measures for Maternal Anxiety

In both studies, we used the Spielberger State-Trait Anxiety Inventory (STAI) to measure maternal anxiety symptoms [24]. The STAI is a reliable and validated self-report questionnaire, consisting of two subscales (state anxiety and trait anxiety), each including 20 items evaluating the level of anxiety. The STAI state anxiety describes the person’s feelings at a specific moment and under particular conditions, whereas the STAI trait scale is used to describe how subjects generally feel. The responses to each item in the anxiety questionnaire are assigned a score from one to four. Possible scores vary from 20 to 80, with higher scores indicating more anxiety. Mothers completed one STAI questionnaire, for both the state and trait domains, upon inclusion in both studies. In the STRONG study, the full STAI questionnaire was additionally completed by mothers upon discharge from the NICU to a high care or medium care unit. In the LPMT trial, mothers filled out the full STAI questionnaire at cross-over in both the LPMT and waitlist group. These procedures are summarized in Figure 1. Our norm population for STAI scores was based on the reference group consisting of working women aged 19–39 years. A normal mean (SD) value of STAI state anxiety was considered to be 36.17 (10.96) and a normal mean (SD) value of STAI trait anxiety was considered to be 36.15 (9.53) [24]. We considered a score of >40 as clinically relevant regarding both STAI state and STAI trait anxiety, as suggested by Dennis et al. [25].

#### 2.3.2. Outcome Measure for Infant Stress

The Neonatal Infant Stressor Scale (NISS), a validated quantitative measure of neonatal stress, was used to measure stress exposure in the infants. The NISS score was developed to quantify the stressful experiences of preterm neonates [26]. It assigns points to various care practices felt by the neonatal care team to be stressful, and classifies these care practices and specific medical events as “acute” or “chronic” stressors. Events are rated on a scale of two to five, in which two is a little stressful, three implies moderately stressful, four very stressful, and a score of five implies an extremely stressful event. For example, for acute items, nappy changes are considered to be moderately stressful, one attempt at gaining intravenous access is very stressful, and multiple attempts to gain intravenous access is extremely stressful. Regarding chronic items, having a nasogastric tube in situ is considered as being a little stressful, fasting for surgery is moderately stressful, and having a systemic infection is very stressful. The NISS does not measure stress experiences by infants, but recently, the cumulative stress exposure has been associated with cortisol measurements, which have proven to be one of the most precise physiological signs of reactions to stress [27]. Therefore, we believe that the NISS is a valid measure of infant stress. During NICU admission, NISS data were abstracted from the medical records of the infants, and the mean NISS score was calculated daily. In the STRONG study, an average NISS score was calculated over days 1 (day of birth) to 14. In the LPMT trial, the average NISS scores were calculated over the total days of the LPMT or waitlist period (Figure 1).

#### 2.3.3. Interventional Music Therapy

Music therapy consisted of a LPMT intervention, provided by a certified neonatal music therapist. During the two-week period of LPMT, infants received six sessions in total. The infants received approximately 15 min of LPMT per session, tailored to their behavioral state and aimed to induce relaxation. Infants in quiet sleep received calmly played music based on entrainment to their breathing patterns. If they were in active sleep, quiet wakefulness, or active states, the improvisation focused on muscle tension and breathing patterns. The musical interventions comprised an acoustic guitar, Ocean Disc, and voice. These were based on the ‘rhythm, breath, and lullaby’ method, which aims to simulate womb sounds and reduce discomfort and stress [28]. An Ocean Disc is a round drum with metal balls that produces sounds as the therapist moves. The Ocean Disc was the primary instrument in the first two sessions. When the guitar was used, the therapist used a song chosen by the parents that was converted into a lullaby (song-of-kin), or the lullaby “Twinkle Twinkle Little Star” [28]. From the third session onwards, song-of-kin was incorporated into the sessions. The music therapist specifically searched for signs of overstimulation in infants. Parents were actively involved and received extensive information on LPMT before the first session, they were mainly present during the sessions, and were provided with their song-of-kin if they had chosen one. A more extensive description of the intervention used in our LPMT trial has been described previously [29].

### 2.4. Statistical Analyses

First, we visually inspected our data using Q−Q plots and tested for normality using the Shapiro−Wilk test. Second, we used descriptive statistics for the demographics of the STRONG and LPMT trial participants. Differences between the two groups of the LPMT trial were tested using the Mann−Whitney-U tests for continuous variables and chi-square tests for dichotomous variables. Third, we compared the STAI state and trait scores of mothers at inclusion and discharge with the reference population and tested differences using independent sample *t*-tests. Next, we assessed the association between STAI state and trait with gestational age and NISS using Spearman’s and Pearson’s correlation coefficients, respectively. To test the effects of LPMT on maternal anxiety, we calculated a delta STAI score between the score after the treatment period and the score at inclusion, and tested differences with an independent sample *t*-test. We decided post hoc to also test the differences in raw STAI scores between the time of inclusion and after a period of either LPMT or waitlist in these groups separately using paired sample *t*-tests. Finally, to incorporate the NISS score in our LPMT trial analyses, we tested the difference in NISS between the LPMT and waitlist groups using independent sample *t*-tests. The level for significance was set at *p* < 0.05 for all of the analyses. Statistical analyses were performed with SPSS (Chicago, IL, USA, version 23) for Windows.

## 3. Results

### 3.1. Participants Characteristics

#### 3.1.1. Characteristics of Participants in the STRONG Study

In total, 93 infants were eligible to participate in this study. Of these, 45 were eventually included. In total, 48 infants were excluded because of declining to participate (*n* = 1), insufficient knowledge of the Dutch language (*n* = 2), no informed consent due to logistical reasons and/or COVID-19 research stop (*n* = 25), and death prior to consent (*n* = 6). In Table 1 we provide an overview of the patient characteristics of the STRONG study participants. The infants had a median gestational age of 27 weeks, and approximately half of the infants were male. Two infants deceased during the study period.

#### 3.1.2. Characteristics of Participants in the LPMT Trial

**Figure 2:**
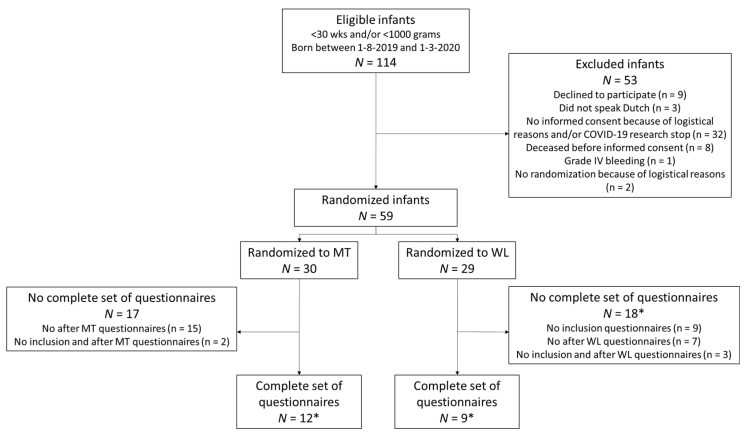
Flow chart of participants in the LPMT trial. LPMT—live-performed music therapy; WL—waitlist. * Including one set of twins.

The flow of the participants in the LPMT trial is presented in Figure 2. In total, 114 infants were eligible to participate in the trial. The parents of 59 infants gave informed consent and these infants were randomized to either LPMT (30 infants) or the waitlist (29 infants). A complete set of questionnaires, i.e., at both the time of inclusion and at cross-over (LMPT-trial), was obtained from 12 out of 30 (40%) infants randomized to LPMT, and from 9 out of 29 (31%) infants randomized to the waitlist. In both groups, the infants had a median gestational age of 27 weeks. The characteristics of the infants in both groups were comparable, except for a significantly higher number of infants with hemodynamically significant persistent ductus arteriosus in the waitlist group. Two infants in the LPMT group died during the study period, including a female twin. Of note, another female twin died on day six, which was before inclusion for the LPMT trial and therefore was not mentioned under the participants’ characteristics. In Table 1 we provide an overview of the patient characteristics of the LPMT trial participants, split by their randomization group. Twelve infants participated in both the STRONG study and the LPMT trial, of which six were randomized to waitlist and six to LPMT.

### 3.2. Maternal Anxiety during NICU Stay and Association between Infant Stress and Maternal Anxiety Symptoms

**Figure 3:**
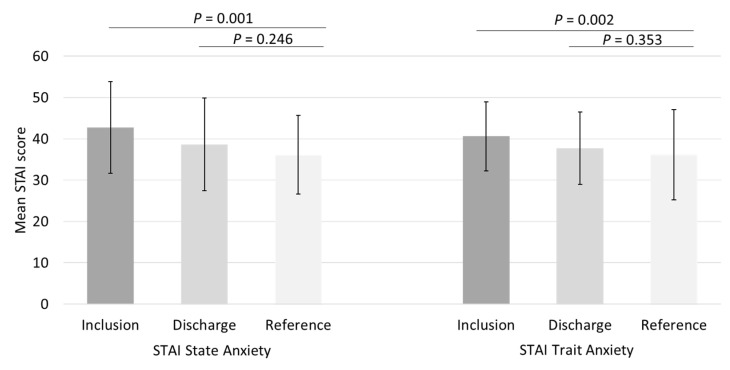
Maternal State and Trait anxiety scores 2 weeks after birth and at discharge, compared with the reference population of working women aged 19–39 years. STAI—State and Trait Anxiety Inventory.

In total, 40 STAI state and 38 STAI trait questionnaires were completed at the time of inclusion in the STRONG study, while 28 STAI state and 27 STAI trait questionnaires were completed upon discharge. During the early NICU stay, both maternal mean (SD) state anxiety and trait anxiety were significantly increased compared with the norm population (42.75 (11.08) vs. 36.17 (10.96), *p* = 0.001 and 40.61 (8.39) vs. 36.15 (9.53), *p* = 0.002), which is shown in Figure 3. At discharge, maternal state anxiety and trait anxiety were comparable with the reference group (38.68 (11.18) vs. 36.17 (10.96), *p* = 0.246 and 37.74 (8.74) vs. 36.15 (9.53) *p* = 0.353).

The higher the cumulative NISS score, the higher the state and trait anxiety scores were, with a strong correlation for state anxiety (Pearson’s r = 0.706, *p* < 0.001) and a moderately strong correlation for trait anxiety (Pearson’s r = 0.490, *p* = 0.002). At discharge, these correlation coefficients were no longer statistically significant (Pearson’s r = 0.260, *p* = 0.181 and Pearson’s r = 0.279, *p* = 0.052 for state and trait anxiety, respectively). We found no significant correlation coefficients between gestational age at birth and state anxiety at inclusion (Spearman’s rho = −0.248, *p* = 0.122), trait anxiety at inclusion (Spearman’s rho = −0.92, *p* = 0.582), state anxiety at discharge (Pearson’s r = 0.274, *p* = 0.159), or trait anxiety at discharge (Pearson’s r = 0.167, *p* = 0.405).

### 3.3. Effects of Our Music Therapy Program on Maternal Anxiety

**Figure 4:**
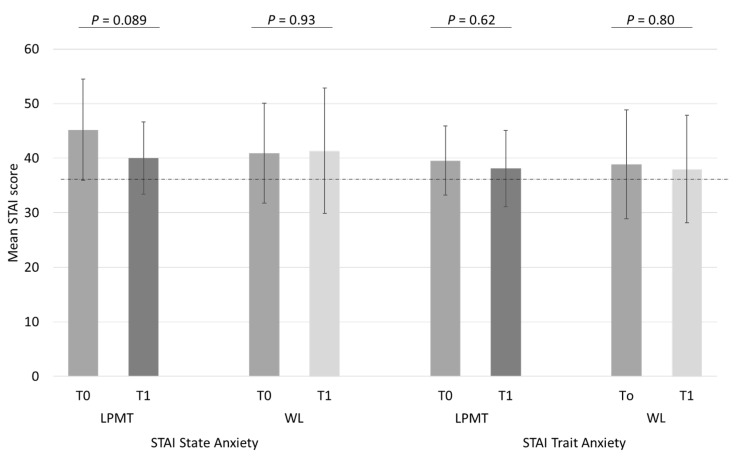
Differences in state and trait anxiety before (T0) and after (T1) either LPMT or waitlist periods. STAI—State and Trait Anxiety Inventory; LPMT—Live-Performed Music Therapy; WL—waitlist. Dotted line represents the norm score of 35 according to the reference population of working women aged 19–39, i.e., 36.17 for state and 36.15 for trait anxiety.

In Figure 4, we present the differences in state and trait anxiety in the LPMT and waitlist groups of our LPMT trial. At time of inclusion, the mean (SD) state and trait anxiety were comparable in both groups (state anxiety 45.2 (9.3) vs. 40.9 (9.2), *p* = 0.31 and trait anxiety 39.3 (6.1) vs. 38.7 (9.4), *p* = 0.85 for LPMT and waitlist groups, respectively). State anxiety scores decreased by 12% in the LPMT group, compared with a slight increase of 1% in the waitlist group (*p* = 0.30). Still, in the LPMT group, the mean (SD) difference was 5.2 (9.6) points. We decided post hoc to also study whether this difference would reflect a significant decrease, and found that it just failed to reach statistical significance (*p* = 0.089), while in the waitlist group, the mean (SD) increase of 0.4 (14.8) points was not statistically significant (*p* = 0.93). For trait anxiety, mothers in the LPMT and waitlist groups had scores that were not different, both before and after the two-week period (decreased scores of 3% and 1.8% in the LPMT and waitlist groups, respectively, *p* = 0.90).

**Figure 5:**
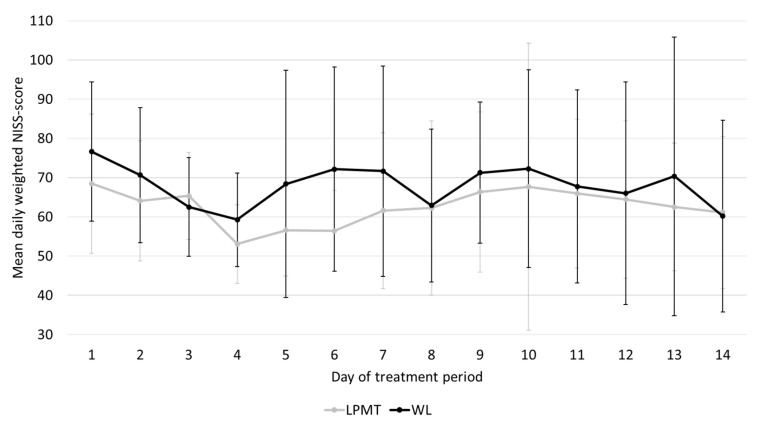
Course of mean daily NISS scores over the LPMT or waitlist periods. LPMT—live-performed music therapy; WL—waitlist.

Infants in the LPMT group had slightly lower mean (SD) NISS scores over their treatment period than the infants in the waitlist group, which was not statistically significant (62.2 (14.4) vs. 68.5 (16.0), *p* = 0.37). For a few infants, the LPMT period lasted longer than 14 days, because of logistical reasons, with a maximum of 20 days. Because the course of NISS scores could also partly explain decreases in maternal anxiety, we calculated the average daily NISS score over the entire 14-day treatment period, and compared these between the two groups. In Figure 5, we show that in both groups, the daily NISS scores declined gradually over time, which did not significantly differ between the LPMT and waitlist groups.

## 4. Discussion

Our study demonstrated that maternal anxiety is elevated early during NICU stay. At the time of discharge, this anxiety decreased to the reference level of working women aged 19–39 years old. We also demonstrated that anxiety in mothers of infants born before 30 weeks of gestation was strongly related to the level of infant stress, and was not affected by the infants’ gestational age at birth. Furthermore, our findings suggested that LPMT may accelerate the reduction of anxiety symptoms in mothers whose very or extremely preterm-born infants were hospitalized in the NICU. LPMT in the first weeks of life thus seems to be a promising and valuable addition to NICU care.

We found that NICU mothers experienced an elevated level of anxiety during early NICU stay, as both state and trait anxiety were significantly increased compared with the reference level of working women aged 19–39 years. Both state and trait levels were >40, meaning this elevated level of maternal anxiety was clinically relevant. This is consistent with the literature [30,31]. Although transition to motherhood itself may be overwhelming and accompanied by feelings of distress [32], in the case of preterm birth, the sudden onset of preterm labor, the abrupt separation from their infant, and fear and uncertainty of how their critically ill infant is doing in the NICU may cause an emotional crisis and negative feelings like anxiety in NICU mothers [33]. In our study, both state and trait anxiety were reduced to normal levels at the time of discharge. This should be interpreted with some caution for two reasons. First, 6 out of the 45 children that participated in the STRONG study had music therapy as they participated in the music therapy arm of the LPMT trial as well. This may have positively influenced the reduction in maternal state anxiety. Second, a considerable part of STAI questionnaires upon discharge were not filled in because of logistical reasons. Nevertheless, our finding corresponds well with Bonacquisti and colleagues who demonstrated that anxiety symptoms decreased two to four months after birth. Despite this decline in anxiety over time, elevated maternal anxiety during NICU stay is reported to be associated with negative outcomes, such as reduced mother−infant attachment [31] and poorer child neurodevelopment [34]. These findings validate the importance of efforts being made to reduce maternal anxiety during early NICU stay.

We found that maternal state, but not trait anxiety, is strongly related to the level of infant stress. To our knowledge, this is the first study that confirms the influence of infant stress on maternal anxiety. We further found that, in mothers of infants born before 30 weeks of gestation, gestational age was not associated with maternal anxiety. This corresponds well with findings by Alkozei and colleagues, who reported that an increased maternal stress level is indeed not so much determined by infant characteristics, such as gestational age, or demographic factors, but much more by parental role alteration [35]. The number of stressful procedures to which an infant is exposed to in the NICU may indicate the extent to which mothers can take on some of the care for their infant themselves. This may explain the negative influence of the NISS score on maternal anxiety by further reducing the parental role. We hypothesize that maternal anxiety diminishes during the NICU stay, not only as parents may get used to their infant being hospitalized, but also because the infant will have to undergo fewer stressful procedures as he or she grows older and stronger. The latter may empower mothers and fathers to grow in their parental role.

In our study, an important reduction in maternal state anxiety was seen after LMPT, compared with the waitlist period. Our results correspond with those of Kehl et al., who found a reduction in anxiety levels in mothers whose preterm infants received creative music therapy [23]. Creative music therapy in their study consisted of humming or singing by a music therapist, preferably in the presence of the parents who had skin-to-skin contact with their child, and was accompanied by vibroacoustic stimulation by an instrument manufactured for therapeutic purposes. Parents could indicate to what extent they wanted to be involved in singing, and their musical and cultural background was integrated into the sessions. The LPMT of the ‘rhythm, breath, and lullaby’ method applies music therapy preferentially based on the vital signs of the infant, incorporating other musical instruments rather than singing alone, and directed to the infant rather than the parent-infant dyad. We demonstrated that this type of LPMT is also effective in reducing anxiety in mothers of preterm-born infants. One explanation may be that seeing an infant in a more relaxed state decreases maternal anxiety. This may confirm the contribution of infant stress to maternal anxiety. Another explanation may be that counseling for our LPMT trial soon after birth provides parents with the confidence to be more involved with their infant’s care in the NICU. Several other studies confirm the positive effects of music therapy on maternal anxiety in the NICU, by providing music therapy to the mother and infant [20,36,37], or to parents alone as part of a self-care group [38]. In contrast, a recent study [39] investigating family centered music therapy for preterm infants could not demonstrate a reduction in maternal anxiety after music therapy intervention. We believe that the late onset of music therapy after the first three weeks of life might be an explanation for the lack of reduction in maternal anxiety in that study. In our study, maternal anxiety was strongly associated with NISS scores, which, in most infants, declined over the first weeks of life, as the infant grows stronger and requires less stressful interventions. We therefore advocate starting music therapy early, to accelerate maternal anxiety reduction during NICU stay. Indeed, Kehl and colleagues reported a significant reduction in state anxiety levels after only six music therapy sessions, which started after the first week of the infants’ life [23]. Interestingly, several studies argue that there may even be a role for music therapy during pregnancy, evidenced by the reduction of maternal anxiety following such interventions [40,41]. This, however, requires further research.

To further strengthen our hypothesis that maternal anxiety decreased because of music, rather than infant stress, we looked for differences between the NISS scores of both groups. The mean total NISS scores of infants during the waitlist period were slightly higher compared with infants receiving music therapy. This might be explained by the higher number of infants with a hemodynamically significant persistent ductus arteriosus in the waitlist group. A hemodynamically significant persistent ductus arteriosus is often associated with a longer duration of mechanical ventilation or nasal continuous positive airway pressure (CPAP), which are both moderately stressful items in the NISS scoring system. Although the course of mean daily NISS-scores did not differ between both groups, the reduction in state anxiety was larger in the LPMT group, which indeed confirms our hypothesis.

The strength of our study was that it confirms the presumed influence of neonatal stress, regardless of gestational age, on the mothers’ wellbeing. Second, we demonstrated that not only creative music therapy [23], but also LPMT based on the ‘rhythm, breath, and lullaby’ method is effective at reducing maternal anxiety in very preterm infants. Third, we used the NISS score to quantify the amount of infant stress, which made it possible to measure cumulative stress exposure. This indicates that it actually is the total amount of stressful stimuli that an infant is exposed to that causes the stress in the mother. We also recognize several limitations. First, we included only a small number of infants in the LPMT trial. The low response after randomization could have caused bias. Stressed parents may not feel the opportunity to complete the questionnaires, as they are occupied with anxiety and worries about their infant’s wellbeing. If it were mainly the less stressed mothers that completed the questionnaires, the found effect of LPMT on maternal anxiety may be an underestimation of the actual effects. Second, we only collected limited background information of the mothers. It would have been interesting to investigate the relation of, e.g., age, maternal socio-economic status, and marital status on anxiety as well. Third, we only focused on the mother−child dyad, as anxiety symptoms seem to be more pronounced in mothers [5]. However, it is known, that, like mothers, fathers of preterm infants also experience more stress than fathers of full-term infants [42]. It would therefore have been interesting to investigate the effects of LPMT on the anxiety of NICU fathers as well. Because music therapy is related to a reduction of anxiety, we believe it may empower early parent−child bonding, which should be confirmed in future research.

The implications of our findings for clinical practice are that all staff, both consulting neonatologists and neonatal nurses, should pay attention to maternal involvement in practical care for their infants. Family integrated and centered care may be a practical framework that can be used by medical staff [43]. Our NICU is an open bay unit, where parents are exposed to more background noise, which may amplify maternal anxiety. LPMT may play an additional role in accelerating a reduction in maternal anxiety, and active involvement of parents in this intervention is crucial for parents and their infants. In The Netherlands, we currently are the only NICU with LPMT. In our country, infants are on the NICU for a relatively short period of time and afterwards are discharged to a high care or medium care ward, where LPMT is also not available yet. We therefore advocate the use of LPMT by a credentialed and specialized music therapist in other NICUs, high care wards, and medium care wards in our country.

## 5. Conclusions

In conclusion, anxiety in mothers of infants born before 30 weeks of gestation is elevated during early NICU stay and is strongly influenced by the infant’s stress, but not by their gestational age. Here, maternal anxiety gradually declined to levels comparable with the reference group at the time of discharge of the infant. Music therapy during early NICU stay may accelerate a reduction in maternal anxiety symptoms because of active involvement and strengthening of mothers in their parental role. Because maternal postpartum anxiety is associated with long-term negative outcomes for both the mother and infant, we recommend integrating music therapy as part of standard NICU care for very preterm infants and their mothers, starting within the first three weeks after birth. Future research should include the effects of music therapy on fathers and parent−child bonding, the child’s development, and its potential role in anxiety reduction during pregnancy, especially in the case of imminent preterm childbirth.

## Figures and Tables

**Table 1:** Characteristics of participants in the STRONG study and LPMT trial.

Characteristic	STRONG (*N* = 45)	LPMT Trial (*N* = 23)	
		LPMT (*n* = 13) ^	Waitlist (*n* = 10) ^
Child characteristics			
Gestational age in weeks	27 (26–28)	26 (25–28)	26 (25–28)
Birth weight (grams)	1000 (790–1248)	900 (703–1045)	810 (734–965)
Male sex	22 (49)	6 (46)	3 (30)
Multiple birth	9 (20)	2 (15) ^&^	1 (10)
Apgar 5 min	7.0 (6.0–8.0)	6.0 (4.5–7.5)	7.5 (5.8–9.0)
NICU admission (days)	34.5 (24–45)	39 (24–48)	53 (37–72)
ACS, ≥1 dose	38 (84)	12 (92)	10 (100)
Sectio caesarea	23 (51)	9 (69)	5 (50)
Mechanical ventilation	30 (67)	9 (69)	9 (90)
Inotropic agents	5 (11)	3 (23)	0 (0)
PDA, hemodynamically significant	20 (44)	3 (23)	7 (70) *
NEC	4 (9)	4 (30)	2 (20)
Surgical NEC	2 (4)	2 (15)	1 (10)
Sepsis, culture proven	16 (36)	2 (15)	3 (30)
IVH ≥ grade III	6 (13)	3 (23)	0 (0)
PVL ≥ grade II	1 (2)	1 (8)	0 (0)
Maternal history			
First pregnancy	27 (60)	6 (46)	6 (60)
Previously premature childbirth (<32 weeks of gestation)	4 (9)	3 (23)	0 (0)
Social and/or psychological problems	7 (16)	0 (0)	1 (10) ^#^

Data are presented as median (25th–75th percentile) or N (%) where appropriate. STRONG—Stress and Outcomes in NICU Graduates; LPMT trial—Live-Performed Music Therapy trial, 12 infants participated in both studies, of which six in the LPMT group and six in the waitlist group; ACS—antenatal corticosteroids; BPD—bronchopulmonary dysplasia; PDA—persistent ductus arteriosus; NEC—necrotizing enterocolitis; IVH—intraventricular hemorrhage; PVL—periventricular leukomalacia. ^ including one set of twins for which only one set of questionnaires was completed; ^&^ one twin sister was deceased before informed consent was obtained; ^#^ i.e., financial problems and involvement of child protection services; * *p* < 0.05 for children in the music therapy versus waitlist group.

## Data Availability

The data presented in this study are available upon request from the corresponding author. The data are not publicly available because of their containing information that could compromise the privacy of research participants.
